# A family-systemic intervention for mental health with refugees in Jordan: Protocol of a randomised controlled trial of StrongerTogether

**DOI:** 10.1017/gmh.2026.10134

**Published:** 2026-02-04

**Authors:** Alexandra H. Blackwell, Hadeel Mansour, Ashraf F. Alqudah, Tamara Jumean, Hadil AlFaqih, Orso Muneghina, Felicity L. Brown, Wietse A. Tol, Mark J. D. Jordans

**Affiliations:** 1 Center for Humanitarian Health, Department of International Health, Johns Hopkins University Bloomberg School of Public Health, USA; 2 Global Health Section, Department of Public Health, University of Copenhagen, Denmark; 3 Research and Development, War Child Alliance, Jordan; 4 Department of Psychology, The University of Jordan; 5 SOS Children’s Villages Jordan, Jordan; 6 SOS Children’s Villages International, Italy; 7 WHO Collaborating Centre for Research and Training in Mental Health and Service Evaluation, Department of Neurosciences, Biomedicine and Movement Sciences, Section of Psychiatry, University of Verona, Italy; 8 Child Protection, United Nations Children’s Fund, United States; 9 Athena Research Institute, Vrije Universiteit Amsterdam, Netherlands; 10 Research and Development, War Child Alliance, Netherlands; 11 Amsterdam Institute of Social Science Research, University of Amsterdam, Netherlands

**Keywords:** randomised controlled trial, process evaluation, global mental health, family systemic, refugees and asylum seekers

## Abstract

Forced displacement heightens mental health risks for children, including psychological, environmental and economic stressors, yet few interventions address whole-family needs within humanitarian contexts. Family-systemic approaches show promise, but evidence on interventions addressing social determinants of mental health remains limited. We will conduct a single-masked, two-arm randomised controlled trial with 550 families in East Amman, Jordan, to evaluate StrongerTogether, a modular whole-family intervention with a financial literacy component. Families experiencing multiple psychosocial challenges will be randomised 1:1 to receive the intervention or enhanced treatment as usual. The trial employs sequential dual outcomes testing, evaluating effectiveness through: (1) upstream improvements in at least one of three primary outcomes (family functioning, parenting practices and caregiver mental health) and (2) direct improvements in adolescent mental health among those with elevated baseline distress. We will also evaluate two implementation tools: ReachNow for family case detection and FamilyACT for facilitator competency assessment. A mixed-methods process evaluation will examine implementation, effectiveness and potential sustainability of core and optional modules. This will be the first rigorous evaluation of an integrated whole-family intervention addressing social and environmental determinants of mental health in humanitarian settings. Findings will inform evidence-based approaches to family mental health support and contribute validated tools for implementation at scale.

## Impact statement

Over 122 million people worldwide are forcibly displaced, with children and families facing devastating mental health consequences that persist long after initial displacement. This research addresses a critical gap by rigorously evaluating a whole-family mental health intervention designed specifically for humanitarian contexts that addresses commonly experienced stressors, including conflict, parenting difficulties, mental health concerns and economic stressors: all major drivers of family distress. Unlike many focused mental health approaches that treat psychological symptoms in isolation, StrongerTogether recognises that refugee families face interconnected challenges requiring integrated solutions. The integration of financial literacy components represents an innovative approach to addressing the root causes of family distress. This holistic model could transform humanitarian approaches to family well-being towards comprehensive support that addresses families’ complex realities. The research will also produce validated tools for identifying families in need and assessing facilitator competencies, practical resources that can be immediately adopted by implementing organisations. For policymakers and practitioners, this research offers evidence-based, accessible mental health support and implementation tools that can be delivered by trained community members, making it sustainable and scalable.

## Introduction

### Background and rationale

By the end of April 2025, 122.1 million people were forcibly displaced worldwide (UNHCR, 2025). Refugees and asylum seekers experience significantly higher rates of mental illness, with lasting impacts on children’s mental health, cognitive development and behaviour (Chen et al., 2019; Blackmore et al., 2020a, 2020b; Arakelyan and Ager, 2021). This situation underscores the critical need for mental health and psychosocial support (MHPSS) interventions for displaced and conflict-affected populations.

Displacement not only impacts children individually (*e.g.*, through experience of traumatic events), it also exposes them to adverse social circumstances, which negatively affect mental health (Scharpf et al., 2021). For example, the fracturing of social and economic structures, combined with harsh living conditions and diminished coping resources, exposes children to increased violence and neglect within their homes and communities (Rubenstein et al., 2020). These environmental stressors undermine family functioning, exacerbating parental distress, family conflict and harsh disciplinary practices (Wessells et al., 2017; Eltanamly et al., 2021). Caregivers struggling with their own mental health challenges may adopt harmful parenting behaviours, creating a cyclical pattern that further compromises child well-being (Saile et al., 2014; Bryant et al., 2018; Rajan et al., 2022).

However, the caregiver–child relationship can also serve as a major protective mechanism, with supportive family environments mitigating against displacement-related adversities. In line with family systems theory, higher family functioning and nurturing relationships have been found to be strong protective factors for both child and caregiver mental health and well-being (Scully et al., 2020). A recent systematic review examining mental health outcomes among forcibly displaced children and families highlights that family cohesion, nurturing parent–child relationships and consistent parental support monitoring can contribute to youth resilience and well-being (Scharpf et al., 2021). This protective effect can also extend to caregivers, with a bidirectional relationship found between caregiver mental health, parenting and child mental health (Pardini, 2008; Sim et al., 2019).

### Whole-family interventions

#### Caregiver- and family-focused interventions

The protective potential of healthy family systems for child outcomes has driven an interest in family-focused interventions in low- and middle-income countries (LMICs) and humanitarian settings (Tol et al., 2011; Giusto et al., 2025). Substantial evidence demonstrates that caregiver-focused interventions can improve parent–child relationships and enhance both caregiver and child mental health across diverse contexts (Vlahovicova et al., 2017; Pedersen et al., 2019; Backhaus et al., 2023; Bosqui et al., 2024). These interventions often work with only caregivers, emphasising caregiver psychoeducation and coping skills development, as well as family-based homework activities.

Significant gaps remain in comprehensive whole-family interventions for mental health in humanitarian contexts. While existing parenting interventions in humanitarian settings effectively reduce violence and improve mental health outcomes (Backhaus et al., 2024), they predominantly address single issues rather than adopting holistic approaches addressing multiple family stressors. The few existing family-focused interventions designed for humanitarian settings rarely address mental health within the broader context of its social determinants, such as economic hardship (Bunn et al., 2022). While direct provision of economic support typically falls outside the scope of focused MHPSS interventions, ‘structure-sensitive’ approaches, or support that acknowledges and works within the broader constellation of family stressors (Patel, 2025), can provide meaningful support through acknowledging families’ real-world concerns and strengthening their coping strategies. This goes beyond most clinical interventions, which only focus on intra-psychic processes (Miller et al., 2021).

#### Addressing social and environmental determinants of mental health

Recent priorities in both research and practice emphasise the need for integrated, multi-sectoral approaches to MHPSS that deliver multilayered systems of care to affected populations to address psychological, social and environmental determinants of health (Lund et al., 2018; Bosqui, 2020; Tol et al., 2023; Giusto et al., 2025). Despite this recognition, the majority of existing MHPSS interventions in LMICs focus on prevention and response at the individual and interpersonal level (*e.g.*, integrating cognitive behavioural therapy and parenting education), failing to address environment-level drivers of poor mental health (Pedersen et al., 2019; Bosqui et al., 2024). Evidence on effective, integrated interventions in humanitarian settings remains particularly limited. While several evidence-based interventions targeting interpersonal violence against children and gender-based violence have integrated MHPSS components into caregiver- and family-focused interventions for conflict-affected families (*e.g.*, Puffer et al., 2017; El-Khani et al., 2021; Sim et al., 2021; Hillis et al., 2024), very few economic programmes have integrated MHPSS components (*e.g.*, Glass et al., 2020; Moyano et al., 2024). Notably, no focused humanitarian MHPSS programmes to date have integrated economic or financial programming. This gap persists despite increasing uptake of the Minimum Services Package for MHPSS (Inter-Agency Standing Committee [IASC], 2022), which demonstrates the demand for the integration of MHPSS across humanitarian sectors. This presents an opportunity for MHPSS interventions to adopt structure-sensitive approaches that, while not providing direct economic assistance, can acknowledge financial stressors as part of the family’s social and environmental context and offer practical skills for financial management within constrained resources. This is a step toward more systematically addressing stressors or social determinants of mental health.

#### Implementation challenges and the need for standardised tools

Notwithstanding growing evidence for the effectiveness of family-focused MHPSS interventions in humanitarian settings, significant challenges remain in translating research findings into scalable, real-world implementation. Key barriers to increasing the reach and adoption of mental health interventions when scaling beyond controlled trial conditions affect both supply and demand. For example, training and supervising sufficient non-specialist facilitators while maintaining quality standards (Troup et al., 2021), and difficulties identifying and engaging families where mental health literacy, help-seeking behaviours and services may be limited (Kazdin, 2019).

The development of standardised implementation tools represents a critical gap in MHPSS research and practice (Giusto et al., 2025). Individual- and community-level case detection tools have demonstrated accuracy in identifying children and adults needing mental health care (*e.g.*, Jordans et al., 2017; Subba et al., 2017; van den Broek et al., 2021, 2023); however, family-level case detection tools have yet to be validated (Brown et al., 2024). Similarly, while validated competency assessment frameworks have been applied in several caregiver-focused (Martin et al., 2021) and psychological interventions for adults and children (Kohrt et al., 2025; WHO, 2025), structured approaches to assess competencies for working with whole families remain underdeveloped (Brown et al., 2022). The rigorous evaluation of implementation tools alongside intervention effectiveness represents an important contribution to implementation science (Proctor et al., 2023), potentially improving both the quality and scalability of family-focused MHPSS interventions in humanitarian settings.

#### Developing a whole-family approach for refugee families in the Middle East

To address these critical gaps, StrongerTogether, a whole-family intervention intended to improve family-system interactions (‘family-systemic’), was developed to improve family functioning and adult and child mental health. The intervention consists of seven core sessions and four optional modules that aim to match the specific concerns raised by individual families (Bosqui et al., Under review).

Previously, a fully powered randomised controlled trial (RCT) evaluating core sessions of the intervention with 351 refugee families in Lebanon demonstrated significant improvements in family functioning, adolescent well-being, caregiver mental health and positive parenting practices (Brown et al., 2022; Bosqui et al., Under review). Next, a feasibility RCT evaluating the intervention consisting of the core sessions plus three optional modules selected by each family was conducted with Syrian, Iraqi and Jordanian families (*n* = 60 families) to confirm the intervention’s feasibility, acceptability and relevance, as well as feasibility of study procedures (Brown et al., 2024). Following this feasibility trial, recognising the profound impact of economic stressors on families’ well-being, the final iteration of StrongerTogether included an optional financial literacy component, which was developed and tested through a pilot case study (*n* = 20 families) as part of the feasibility trial. This progressive development reflects a commitment to addressing the complex, interrelated challenges faced by families in humanitarian contexts through a holistic, modular approach impacting immediate psychological needs while being sensitive to underlying social determinants of mental health. Evidence of the effectiveness of the core intervention and its optional components through a fully powered RCT is now needed.

### Objectives and hypotheses

The primary aim of this study is to evaluate the impact of a whole-family intervention on social and contextual determinants of adolescent mental health (family functioning, parenting behaviours and caregiver distress) and subsequently adolescent mental health problems among forcibly displaced and socio-economically vulnerable families living in Jordan. Given that the intervention is intended to impact the entire family system, we will use a sequential testing approach to evaluate the effect on outcomes at multiple levels (*i.e.*, for different family members). Secondarily, it will evaluate the adapted versions of two tools for implementation of whole-family interventions for mental health: FamilyACT for assessing facilitator competency, and ReachNow for proactively selecting families in need of focused, non-specialist mental health support. The process evaluation aims to illuminate the effectiveness of the core and optional modules and mechanisms of change in addition to assessing their implementation and potential sustainability.

#### Primary effectiveness hypotheses

StrongerTogether will demonstrate effectiveness through sequential testing of dual pathways:

1. H1 (upstream mechanisms): Families receiving StrongerTogether will show significantly greater improvements in at least one upstream determinant (family functioning, parenting practices or caregiver mental health) compared to enhanced treatment as usual (ETAU) families at endline (T2).
2. H2 (direct effects in high-need families): Among families with adolescents showing elevated baseline distress (emotional and/or behavioural difficulties), the intervention will demonstrate superior effectiveness in reducing adolescent distress compared to ETAU at endline (T2).
3. H3 (moderation effect on downstream outcome): The intervention will strengthen the protective relationship between upstream improvements and adolescent mental health, such that treatment families demonstrate greater child mental health benefits from upstream improvements in family functioning, parenting practices or caregiver mental health compared to control families at endline (T2) and follow-up (T3).

The study is fully powered to test H1 (power = 0.85). H2 and H3 are positioned as exploratory analyses, given limitations regarding sample size (see below for sample size calculation).

#### Primary implementation hypotheses

The ReachNow tool will accurately identify 70% or more of families in need of focused mental health support as identified through screening results. In addition, the FamilyACT tool will demonstrate adequate reliability and validity for assessing facilitator competencies in delivering family-systemic interventions in humanitarian settings.

The study will secondarily explore mechanisms of change of the intervention through mixed-methods exploratory longitudinal analyses.

### Trial registration

The study will be prospectively registered with ISRCTN in accordance with the Standard Protocol Items: Recommendations for Interventional Trials 2025 checklist (Chan et al., 2025).

## Methods

### Study setting

The study will take place in East Amman, Jordan. Jordan hosts the second largest population of refugees *per capita* globally, with an estimated 1.3 million refugees and asylum seekers (UNHCR, 2024a) in a total population of ~11 million (United Nations Jordan, 2022). Over 80% of the refugees in Jordan have settled in urban and peri-urban areas outside of formal camp settings (UNHCR, 2024b). East Amman is one of the most densely populated areas in Jordan, with the neighbourhood of Hashmi Shamali home to over 200,000 refugees from six different countries as of May 2025.

Many refugees in Jordan experience severe psychological distress, with two-thirds meeting symptom criteria for depression, anxiety or posttraumatic stress disorder (Nazzal et al., 2024). Displacement-related distress is exacerbated by overlapping stressors, including insecure income and housing, inaccessible services, disrupted social support, exploitation and discrimination (Wells et al., 2016; Chen et al., 2019; Barrett et al., Under review). The burgeoning humanitarian population has overwhelmed public systems within Jordan, where long-term regional instability and economic hardship have diminished the country’s health, education and economic systems. This has resulted in a high level of unmet need for MHPSS for populations facing chronic adversity. Jordan’s Ministry of Health emphasised the need for family-based psychosocial support within East Amman, specifically (World Health Organization, 2024).

This study is a partnership between War Child Alliance, SOS Children’s Villages Jordan, University of Copenhagen, and the University of Jordan. Both StrongerTogether and ETAU will be implemented by two community-based organisations: the Families Development Association in Al Hashmi Al Shamali and the Nawafez Al Ata’ Association (Windows of Giving) in Tabarbour, near the Al Hashmi Al Shamali area.

### Trial design

The study is a single masked, two-arm RCT with an embedded qualitative process evaluation. Families will be randomised to the intervention or ETAU arms in a 1:1 allocation ratio. Outcomes will be assessed at baseline prior to randomisation (T0), at midline after the completion of core sessions (T1), at endline after the completion of advanced modules (T2, primary time point) and at follow-up 6 months after endline (T3).

The trial employs a sequential, dual outcomes approach to establish effectiveness through multiple pathways comparing treatment *versus* control conditions: (1) upstream improvements in family functioning, parenting practices or caregiver mental health in the full sample; (2) direct improvements in mental health in high-distress adolescents; or (3) enhanced protective effects of upstream improvements on adolescent mental health. Secondary exploratory analyses will examine mechanisms of change over time and the additive effect of the optional modules compared to core modules.

The embedded process evaluation will evaluate implementation, effectiveness and potential sustainability of core and optional modules through mixed methods, including surveys, programme monitoring, staff debrief meetings and qualitative interviews and focus groups. Qualitative activities will be conducted post-intervention (T2) with families who complete and drop out of the intervention, facilitators, outreach staff and other key personnel.

### Intervention and control conditions

#### Whole-family intervention: StrongerTogether

Intervention characteristics are reported in accordance with the template for intervention description and replication (TIDieR) checklist (Hoffmann et al., 2014). StrongerTogether is a modular, whole-family intervention addressing multiple interconnected family challenges simultaneously. It is positioned as a Level 3 intervention on the IASC MHPSS pyramid,^1^ providing focused, non-specialised mental health support for individual families with psychological distress (IASC, 2007). Non-specialised services rely on task-sharing, an evidence-based approach involving the delivery of mental health services by trained providers who do not have specialised or formal mental health training, improving accessibility (Kazdin, 2019; Raviola et al., 2019).

For each family, the intervention is delivered to at least one caregiver and all adolescents aged between 10 and 17 years. It comprises seven core sessions (including six content-specific sessions and a collaborative ‘transition’ session) addressing family roles, communication, problem-solving and future planning, followed by four optional modules of up to four sessions each covering parenting, caregiver mental health, disagreements and financial literacy, as needed. Session content is manualized, a detailed overview of which can be found in Table 1. Sessions take place weekly at a community centre and are delivered by one non-specialist facilitator face-to-face with individual families, with the support of an assistant to coordinate logistics and provide activities for younger children who may attend but not engage in the entire session. Each session involves a 90-min guided activity or discussion with the entire family, followed by a 30-min discussion with caregivers alone to consolidate the information and support them in their role to implement the strategies. To facilitate home practice and sharing of content with non-attending family members, session content is summarised and provided to families through simple, illustrated handouts and audio-recordings created by the StrongerTogether master trainer to accommodate varying literacy levels.

**Table 1.** Module content and aims

Central family intervention module		Number of sessions
Module	Aims	
1 Our family story	Family develops joint understanding of challenges, goals and strengths of the family Family develops joint understanding of roles and responsibilities Caregivers are encouraged to work together as a team	7
2 How our family wants to be	Family develops a joint understanding of challenges, goals, and strengths of the family Family understands each other and build empathy	
3 Managing difficult feelings and helping each other	Family members build awareness of feelings experienced by themselves and others Family builds skills in grounding and stepping back from thoughts to help them manage difficult thoughts and feelings Family learns how to practice good self-care for positive well-being	
4 Communicating well to support each other	Family understands their common barriers to positive communication Family builds skills in positive communication, including good listening, and expressing themselves	
5 Managing our problems together	Family identifies common problems they are facing Family learns and applies the Stop Think Go problem management steps to their problems	
6 Managing problems when we do not all agree	Family discusses strengths, roles and responsibilities in the family Family identifies common sources of conflict Family learns and applies the Stop Think Go problem-solving steps to problems involving disagreements between family members	
7 Reviewing where we started and where we want to go in the future	Family reviews content learned in sessions Family reviews home practice of strategies and troubleshoots any challenges they have faced Family makes a plan for the future, maintaining progress made through these sessions Facilitator collaboratively determines with caregivers whether they will complete additional modules	

Optional modules		
1 Parental support	Caregivers develop self-management strategies for challenging parenting issues Caregivers apply problem management skills to specific parenting challenges Caregivers build skills to establish Family Rules, using directed praise and modelled behaviour	2–4 (Caregivers only)
2 Managing difficult thoughts and feelings	Caregiver and facilitator develop a joint understanding of current challenges, triggers and goals Caregiver understands how avoidance behaviours can worsen difficult thoughts and feelings 3. Caregiver learns to disengage from difficult thoughts and feelings	2–4 (Caregivers only)
3 Support managing disagreements	Family works on current disagreements using skills from core sessions Family strengthens the ability to apply communication and problem management skills to disagreements	1–2
4 Financial management challenges	Family understands financial literacy concepts and their importance for family stability Family learns to identify financial needs, create budgets and establish emergency savings Family develops skills in responsible spending, debt management and regular saving habits	3

While facilitators are not mental health specialists, they will be selected based on past experience conducting MHPSS activities and working with children, adults and families in the community. All facilitators receive 10 days of training following a structured curriculum introducing core and advanced module content, facilitation techniques, psychological first aid, child safeguarding, ethics and confidentiality and competencies for working with families. Training workshops are conducted by a local experienced MHPSS supervisor. The supervisor provides weekly group supervision sessions to facilitators, covering challenges and successes during implementation, facilitator competencies, management of adverse events and referrals and self-care techniques.

#### Enhanced treatment as usual (ETAU)

The control condition will be an ETAU consisting of one 30–45 min session comprising a structured, family psychoeducation session delivered by a non-specialist facilitator to the participating caregivers and index adolescent. The scripted session consists of (1) sharing the screening results with the family; (2) discussing common and simple self-care strategies to manage distress; and (3) providing information about available MHPSS, caregiving and financial literacy resources and services. The ETAU will be delivered by non-specialist facilitators, who are different from the StrongerTogether facilitators and who receive a separate 1-day training on the delivery of the psychoeducation session.

### Participants

#### Sample population

Participants in the study will be forcibly displaced and host families living in three districts in East Amman, Jordan, that are vulnerable due to a combination of mental health, family and economic factors. For the purposes of this study, we use the term ‘forcibly displaced’ to refer to refugees, asylum seekers and individuals and families of varying legal status who have left their country of origin and are living in Jordan. Families can be of any size or composition (*i.e.*, single-headed, dual-headed and mixed generations), provided there is a legal guardian able to provide consent and take part in the intervention and study. Sampling will be conducted using a community-based case detection and screening approach to ensure that we recruit families most in need of focused mental health support.

#### Participant inclusion/exclusion criteria

Families are eligible to participate in the study if they have at least one child aged 10–17 years and are experiencing two or more psychosocial challenges (where condition A and/or B exists alongside at least one condition from C to E). A brief screening interview will be used to assess the family’s eligibility for the intervention, including:

- At least one caregiver has mental health challenges;
- At least one child has mental health challenges;
- Family relationship challenges causing significant distress or impairment;
- Caregiver parenting challenges causing significant distress or impairment; or
- Family financial stress.

The screening tool will also include two questions to assess the risk of harm through participation.

For a family to participate, all caregivers must provide consent, and at least one child aged 10–17 years must assent to take part. Participants will be excluded from the study if any member of the family:

1. Do not consent or assent;
2. Indicate safety concerns or reluctance to take part in a whole-family programme during screening;
3. Are unaccompanied minors or children who are married (due to guardian consent challenges);
4. Have severe psychiatric, substance abuse or other conditions requiring specialist services (assessed through elevated scores on screening tools);
5. Have a severe disability impacting participation that cannot be overcome with reasonable accommodation, thereby requiring specialist services; or
6. Any family member already receiving Level 3 focused MHPSS (*i.e.*, more targeted care for children or adults with higher levels of distress; for example, those that screened positive on a symptom scale) or Level 4 specialist mental health interventions (IASC, 2007).

Cases requiring specialised services will be referred to the appropriate actors in each location, as this Level 3 intervention is not suitable for severe mental health conditions or protection concerns requiring specialist support.

#### Sample size

The sample size calculation was powered for the sequential dual outcomes approach. Assuming a statistical significance level of 5% with an intra-cluster correlation coefficient of 0.02 and a two-tailed test with 0.85 power, a total sample of 440 index adolescents and 440 caregivers is required (220 per group) to detect a minimum effect size of *d* = 0.15 for any of the three upstream primary outcomes (family functioning, parenting practices or caregiver mental health) (Brown et al., 2022, 2024). This is based on conservative estimates consistent with our previous trials with similar populations (Brown et al., 2022) and the feasibility study (Brown et al., 2024). To account for an anticipated 20% attrition rate due to contextual challenges frequently encountered in humanitarian settings, as well as sub-analyses based on family composition and uptake of advanced modules, the study will aim to recruit between 550–600 index adolescents and their families.^2^

### Procedures

#### Recruitment, informed consent and data collection

##### Pre-study evaluation of ReachNow detection tool

To recruit families, outreach staff will be trained to use an adapted version of ReachNow, a proactive community case detection tool (van den Broek et al., 2023). The proactive case detection approach employs illustrated vignettes depicting a family experiencing multiple psychosocial challenges (including mental health difficulties, family conflict and financial stress). The simple decision algorithm asks outreach staff to assess whether the depicted family resembles families they know in their community who might benefit from focused mental health support. If they identify similarities, they are prompted to refer those families for assessment. Previous versions of the child-focused tool demonstrated 70% accuracy compared to clinical assessment in Sri Lanka and Palestine (van den Broek et al., 2021, 2023). The family-adapted version was piloted in Jordan during the feasibility study with 78 families, demonstrating feasibility and appropriateness. In the present study, the tool will be formally evaluated for accuracy.

War Child Jordan will identify and train outreach staff on the participant inclusion/exclusion criteria and how to use the family version of the ReachNow detection tool to identify families who are eligible for the intervention. These individuals are trusted and respected community members who have established and regular communication with families who are struggling, including personnel and volunteers. During outreach for study enrolment, the family version of the ReachNow tool will be evaluated for accuracy by comparing the ReachNow caseness with screening outcomes assessing mental health challenges (caregiver and child), family functioning difficulties, parenting challenges and financial stress in accordance with study inclusion criteria.

##### Recruitment

Recruitment will be led by outreach staff under the supervision of War Child Jordan during the study enrolment period using the family version of the ReachNow detection tool. Outreach staff will contact families who meet the eligibility criteria, inform them about the intervention using a script and offer an invitation to participate. Those families who confirm interest will be asked for verbal consent and then referred to the research team for the screening assessment.

##### Informed consent and assent

Informed consent procedures will involve providing families with both oral and written information about the study. Trained research assistants will read the consent form to families and provide opportunities for questions. For families who agree to participate, signed consent will be sought from both caregivers where applicable (with an option for witnessed verbal consent), as well as verbal assent from participating children. Particular care will be taken to ensure understanding of voluntariness, and participants will be informed they can decline or withdraw from the study at any time without affecting their ability to participate in the intervention or receive services from the implementing organisations.

##### Screening

Screening assessments will be administered to caregivers by trained research assistants using tablets in private locations, with family members assessed one-by-one. The screening protocol will assess families for: caregiver mental health challenges (Kessler-10 [K10] ≥ 20); child mental health challenges (Strengths and Difficulties Questionnaire [SDQ] caregiver report ≥17); family relationship challenges (screening questions developed for study); caregiver parenting challenges (screening questions developed for study); financial stress (screening item developed for study); and absence of risks. One caregiver will be asked to report on children in the household. In households with more than three children, they will be asked to report on the children with the most severe emotional and behavioural challenges. Based on the caregiver-reported screening assessment for child mental health challenges, one index adolescent will be selected for subsequent data collection. If more than one child aged 10–17 years scores above the cutoff on the SDQ (≥17), the highest scorer will be selected as the index adolescent for the purposes of the study. Families who meet eligibility requirements will proceed to complete the baseline assessment. More details on the screening protocol, including cut-offs, can be found in Table 2 and Supplementary Document A.

**Table 2.** Screening protocol for study eligibility

Criterion	Inclusion criteria	Contextual validity
1a. Caregiver mental health	At least one caregiver scores ≥20 on the K10	Cut-off validated for detecting serious mental illness in general populations (Kessler et al., 2003) and feasibility study (⍺ = 0.85) (Brown et al., 2024)
1b. Child mental health challenges	At least one child scores ≥17 on the caregiver-report SDQ	Cut-off validated for detecting serious emotional and behavioural problems in general populations and acceptable internal consistency (⍺ = 0.73) in Arabic versions of parent- and teacher-report (Ababneh and Alomari, 2016; Emam et al., 2016; Al-Hendawi, 2023)
1c. Family relationship challenges	Structured screening questions were developed for this study and piloted in the feasibility study, assessing the presence of significant family conflict or dysfunction causing distress or impairment	Developed for context and validated in a feasibility study
1d. Parenting challenges	Structured screening questions were developed for this study and piloted in the feasibility study, assessing parenting difficulties causing significant distress or impairment	Developed for context and validated in a feasibility study
1e. Financial stress	Structured screening questions were developed for this study and piloted in the feasibility study, assessing family financial difficulties causing significant distress or impairment	Developed for context and validated in a feasibility study
2. Absence of severe risks requiring specialist care	Structured screening questions about serious concerns where at least one caregiver indicates potential for harm (*e.g.*, suicidality, severe substance abuse, active psychosis, ongoing family violence, *etc.*) Observed or reported challenges for any family member with suicidality, disordered thinking, severely impacted functioning, or other symptoms of serious mental health concerns, as well as serious protection concerns such as intimate partner violence, child abuse or coercion	Structured questions developed for context and validated in feasibility study Observation or disclosure during screening or data collection

##### Data collection

Research assistants will collect survey data from at least one caregiver and the index adolescent at baseline (T0: no more than 3 weeks before intervention), midline (T1: post-core modules), endline (T2: post-optional modules, within 3 weeks of the final session) and 6-month follow-up (T3). Surveys will collect sociodemographic information, outcome measures and hypothesised mediators (see below). After baseline, intervention families will be contacted to schedule sessions. Families discontinuing the intervention will be encouraged to complete the remaining assessments.

Research assistants will collect qualitative data using semi-structured guides after endline from a purposive subsample (*n* = 15) to examine implementation, effectiveness and potential sustainability. At least 10 families who completed the intervention and five who dropped out will be selected for interviews, ensuring representation across family composition, attendance and change in outcomes at T1 and T2. Focus group discussions (*n* = 4) and key informant interviews (*n* = 8) will be conducted with stakeholders from implementing organisations, including facilitators, supervisors and other staff, to explore implementation factors, programme sustainability and recommendations.

#### Randomisation

Randomisation will occur after baseline assessments. Families will be randomly allocated to treatment or ETAU conditions in a 1:1 ratio. A computer-generated randomisation sequence will be created by an independent statistician external to the study location using Research Randomizer (randomizer.org). Complete, temporal participant lists will be provided to the independent statistician, who will match them to the randomisation sequence and return allocations for implementation.

#### Masking

All investigators and research assistants will be masked to the allocations of families. Due to the nature of the intervention, complete masking of participants and implementation staff is not possible. Staff will receive training on maintaining masking protocols during data collection, research and implementation teams will operate separately, and participants will be instructed not to reveal their allocation. Research assistants will document instances where allocation is accidentally revealed. The level of (un)masking of the research assistants will be assessed at the end of each follow-up interview by asking research assistants if allocation was explicitly revealed during the interview. These data will be used to assess the integrity of masking and to control for possible bias in impact analyses, in addition to being reported as a study limitation.

### Outcomes

Outcome measures were selected based on prior validation with similar populations in Arabic. During the feasibility study, measures underwent a multi-step translation process (*i.e.*, forward and backtranslations by independent bilingual team members, translation workshops and cognitive interviewing) and psychometric evaluation to ensure validity and relevance for the target population (Brown et al., 2024). The instruments can be found in Table 3.

**Table 3.** Key effectiveness and implementation measures

Construct	Measure	Scale construction	Contextual validity
***Primary upstream outcomes***			
Family functioning (adolescent-report)	Adapted Systemic Clinical Outcome and Routine Evaluation–15 (SCORE–15) (Stratton et al., 2010)	16-item measure (15 + 1 added) (range: 16–80) 5-point Likert scale rating applicability to family Covers strengths and adaptability, feeling overwhelmed by difficulties and communication quality Lower score indicates better functioning	Very good internal consistency (⍺ = 0.90) in feasibility study (Brown et al., 2024); good psychometric properties with similar populations (Hamilton et al., 2015)
Parenting practices (caregiver-report)	Arabic Dimension of Parenting Scale (Miller et al., 2020)	25-item measure (24 + 1 added) (range: 25–75) 3-point Likert scale (rarely, sometimes, often) Assesses parental warmth and responsiveness, positive parenting, and harsh discipline practices Higher scores indicate more positive parenting	Very good internal consistency in feasibility study (⍺ = 0.88) and in trial with psychometric testing of Arabic version used with Syrian refugees (⍺ = 0.87) (Miller et al., 2020)
Caregiver mental health (caregiver-report)	Kessler–10 (K10) (Kessler et al., 2003, 10)	10-item brief screening scale (range: 10–50) 5-point Likert scale Higher scores indicate more distress	Good internal consistency (⍺ = 0.85) in the feasibility study
***Primary downstream outcome***			
Adolescent emotional and behavioural difficulties (adolescent-report)	Strengths and Difficulties Questionnaire (SDQ) (Alyahri and Goodman, 2006)	25-item measure (range: 0–40) 3-point frequency scale (not true, somewhat true, certainly true) Covers emotional problems, conduct problems, hyperactivity, peer problems and prosocial behaviours Higher scores indicate greater difficulty	Good internal consistency (⍺ = 0.73–85) and psychometric testing of both child- and adult-report Arabic versions with similar populations (Ababneh and Alomari, 2016; Emam et al., 2016; Atoum et al., 2018; Al-Hendawi, 2023)
***Caregiver-reported secondary outcomes***			
Family functioning (caregiver-report)	Adapted Systemic Clinical Outcome and Routine Evaluation–15 (SCORE–15)	Same as adolescent-report (above)	Very good internal consistency (⍺ = 0.89) in feasibility study
Financial literacy and capability (caregiver-report)	Financial knowledge, behaviours, decision-making skills and well-being	Measure developed for the present study	Similar measures of financial literacy, capability and well-being have been used with populations in Jordan (Alqam and Hamshari, 2024)
Emotional regulation (caregiver-report)	Adapted Difficulties in Emotion Regulation Scale 18 (DERS–18) (Victor and Klonsky, 2016)	19-item measure (18 + 1 added) (range: 19–95) 5-point Likert scale assessing frequency (1 = almost never; 5 = almost always) Assesses emotional awareness, acceptance, goal-directed behaviour and regulation strategies Higher scores indicate more difficulties	Very good internal consistency (⍺ = 0.87) in feasibility study
Mental well-being (caregiver- and adolescent-report)	Warwick-Edinburgh Mental Wellbeing Scale (WEMWBS) (Stewart-Brown and Janmohamed, 2008)	14-item measure (range: 14–70) Items rated on a 5-point scale Assesses positive mental health and well-being	Acceptable internal consistency (⍺ = 0.72) in trial with Syrian refugees (Miller et al., 2020)
Adolescent emotional and behavioural difficulties (caregiver-report)	Proxy-reported SDQ (Goodman, 2001)	25-item measure (range: 0–40) 3-point frequency scale (not true, somewhat true, certainly true) Covers emotional problems, conduct problems, hyperactivity, peer problems and prosocial behaviours Higher scores indicate greater difficulty	Acceptable internal consistency (⍺ = 0.73) and psychometric testing of parent- and teacher-report Arabic versions with Arabic-speaking adolescent populations (Ababneh and Alomari, 2016; Emam et al., 2016; Al-Hendawi, 2023)
Adolescent self-perception and belief in personal goal attainment (adolescent-report)	Children’s Hope Scale (Snyder et al., 1997)	6-item continuous measure (range: 0–3) averaging the total responses 3-point Likert scale rating how often they think/feel each item, ‘Almost never’ to ‘Most of the time’ Higher score suggesting higher perceived hope	Very good internal consistency (⍺ = 0.93) in Jordan (O’Leary et al., 2015); good psychometric properties with forcibly displaced adolescent populations in South Sudan (Blackwell et al., 2023), Uganda (Metzler et al., 2022), Burundi, Indonesia and Nepal (Haroz et al., 2017)
***Implementation and process evaluation measures***			
Proactive family-level case detection	ReachNow family version	Illustrated vignettes showing a prototype family experiencing multiple psychosocial and financial challenges Simple decision algorithm to identify families in need of mental health care Used by community outreach staff for family identification	Approximately 70% of children identified using the tool were confirmed in need of mental healthcare in clinical assessment in Sri Lanka and Palestine (van den Broek et al., 2021, 2023); an adapted tool was piloted with 78 families in Jordan during feasibility study
Facilitator competency	Family Act Tool (FamilyACT) (WHO, 2025)	Observational rating tool for MHPSS interventions delivered by non-specialists Developed for the World Health Organization EQUIP (Ensuring Quality in Psychological support initiative) platform Adequate single-rater reliability (ICC: 0.40) Sensitivity to change over time (Wilcoxon signed-rank tests, *α* < 0.05)	Developed for interventions where non-specialists deliver MHPSS interventions (Kohrt et al., 2025); with similar tools used in previous studies with high inter-rater reliability (ICC: 0.71–0.89) (Pedersen et al., 2021; Mwenge et al., 2022), single-rater reliability (ICC: 0.41–0.75) and demonstrated sensitivity to change (Jordans et al., 2021)
Implementation fidelity	Module completion, supervisor observation and programme monitoring and supervision records	Programme monitoring Documentation of supervisor observations Debrief meetings on facilitator module completion and implementation Fidelity will be calculated as the percentage of core components delivered as intended following session observations by the supervisor	NA
Implementation acceptability and potential for scale	Module attendance, acceptability and barriers and facilitators to implementation	Documentation of module completion and session participation per family member, with reasons for non-attendance recorded Qualitative focus groups and interviews	NA
Process documentation	Programme monitoring and debrief notes	Programme monitoring Debrief meetings on progress and challenges with outreach, family module completion and implementation	NA

**Table 4.** Process evaluation outcomes and measures for implementation, effectiveness and potential sustainability by the RE-AIM framework

Dimension	Study definition	Participant level	Organisation level
Reach	Representativeness of the participants who enrol and the individuals’ willingness to participate in the study	Qualitative: Reasons for non-participation or discontinuation Monitoring: Attendance records	Debrief meetings: Barriers to recruitment Quantitative: ReachNow evaluation
Effectiveness	Impact of the intervention on target outcomes, including potential negative effects, heterogeneity of effects and mechanisms of change	Quantitative: Intervention effectiveness; Subgroup analyses; Moderation and exploratory mediation analyses Qualitative: Perceived impact and pathways of change	Monitoring and debrief meetings: Adverse events monitoring Qualitative: Perceived impact and pathways of change
Adoption	Organisational support to deliver the intervention, including acceptance of the intervention among implementing organisations and reasons for (non-)adoption	Quantitative: Moderation of intervention effects by facilitator characteristics	Quantitative: Facilitator competency (FamilyACT) Qualitative: Perceived feasibility, acceptability and sustainability of the intervention
Implementation	Completion of intervention components as intended, as well as acceptability (participants’ use of the intervention) and potential for scale-up	Qualitative: Acceptability of core and optional modules	Monitoring: Completion of modules Qualitative: Barriers and facilitators to implementation; Recommendations for sustainment and scale
Maintenance	The extent to which a behaviour is sustained and the intervention becomes institutionalised	Not applicable to this study	

#### Upstream mechanisms

*Family functioning* will be assessed using an adapted version of the Systemic Clinical Outcome and Routine Evaluation-15 (SCORE-15) (Stratton et al., 2010), which has shown good psychometric properties in previous studies with similar populations (Hamilton et al., 2015), including adequate internal consistency in the feasibility study (*α* = 0.80) (Brown et al., 2024). One additional item about satisfaction with family decision-making was added during the feasibility study and has been retained. The adapted version of the SCORE-15 will be reported by both adolescents and caregivers, with adolescent-report used to assess primary effectiveness.

*Parenting practices* will be evaluated through the Arabic Dimension of Parenting Scale (caregiver-report), a 24-item measure addressing parental warmth, positive parenting strategies and harsh discipline practices (Miller et al., 2020). One additional item on parental problem-solving was added during the feasibility study and has been retained.

*Caregiver mental health* will be measured with the K10, a brief 10-item scale screening for psychological distress (Kessler et al., 2003), while *mental well-being* will be assessed using the Warwick-Edinburgh Mental Wellbeing Scale (WEMWBS) (Stewart-Brown and Janmohamed, 2008).

#### Downstream adolescent outcomes (index adolescent)

*Adolescent mental health* will be assessed using the SDQ reported by both adolescents (Alyahri and Goodman, 2006) and caregivers (Goodman, 2001). Primary effectiveness will be assessed by adolescent-report.

*Well-being* will also be assessed using the same WEMWBS scale as administered to caregivers, providing parallel measurement across family members, as well as the Children’s Hope Scale (Snyder et al., 1997).

Additional exploratory outcomes, sociodemographic characteristics and implementation data will also be collected and are outlined in Table 3. These variables will also be controlled for in effectiveness analyses to account for variation over time.

#### Implementation tool evaluation

The *accuracy of case detection* of the ReachNow family tool will be validated by comparison against the screening results. Sensitivity, specificity, positive predictive value and negative predictive value of the ReachNow tool will be calculated using the screening results as the standard.

*Facilitator competency* will be assessed using the FamilyACT tool, an observational rating scale based on existing tools within the World Health Organization Ensuring Quality in Psychological Support (WHO EQUIP) platform for assessing competencies of non-specialists delivering MHPSS interventions (WHO, 2025), and adjusted for whole-family interventions during earlier studies in Lebanon (Brown et al., 2022) and Jordan (Brown et al., 2024). The tool assesses common competencies for working with families across 15 domains, with reliability across contexts (Kohrt et al., 2025). Each facilitator will be observed and rated by a trained competency rater at four timepoints: (1) during the training (days 2 or 3), (2) during the post-training practice round, (3) early intervention (sessions 1 or 2) and (4) after completion of the seven core sessions. The reliability of the tool will be assessed by calculating internal consistency (Cronbach’s 𝛼 ≥ 0.70) and single rater-reliability (single measures intraclass correlation coefficient ≥ 0.40) at each time point. The tool’s sensitivity to change will also be evaluated through measurement of facilitators’ competencies across timepoints through statistical testing using Wilcoxon signed-rank tests (*W*) (*α* < 0.05) to evaluate changes in levels of competencies for the newly trained facilitators over time.

### Data management

Survey data will be collected using a secure data collection platform on password-protected tablets and uploaded to a secure, encrypted server accessible only to authorised team members. Qualitative data will be stored on the encrypted server, and audio files will be deleted once transcription and verification are complete. Individuals and families will receive unique identification codes linking responses across time points. A master code sheet linking names to study IDs will be stored separately on a password-protected server, accessible only to core research team members. De-identified data will be stored securely, with statistical code and anonymised data made publicly available following institutional guidelines.

### Statistical analysis

#### Effectiveness analyses

Trial results will be reported following the updated recommendations of the Consolidated Standards of Reporting Trials (CONSORT) 2017 statement for randomised trials of nonpharmacologic treatments (Boutron et al., 2017). All analyses will use an intent-to-treat approach, including all randomised participants regardless of intervention adherence. Statistical significance will be evaluated with a significance level of *p* < 0.05 for each step, with analyses proceeding regardless of preceding step results to fully understand intervention mechanisms.

*Primary effectiveness analyses* will test dual outcome hypotheses sequentially to examine intervention effects on upstream and downstream outcomes using hierarchical linear modelling to account for the nested data structure: (1) repeated measurements within individuals; (2) multiple individuals nested within families; and (3) families randomised to intervention or control conditions. T2 is the primary time point for evaluating intervention effects using group × time interactions, accounting for individual-level fixed effects (*e.g.*, age, gender and baseline measures) and random effects at the individual and family levels (*e.g.*, variation in stress over time).

*Secondary exploratory analyses* will test moderation effects and employ latent growth curve modelling to investigate treatment response patterns and mechanisms of change. Sub-group analyses will examine differential effects by facilitator characteristics, module completion and family and individual characteristics, including time since displacement. Sensitivity analyses will include per-protocol analyses limited to participants completing sufficient dosage of core sessions and alternative missing data approaches.

#### Process evaluation

Informed by the Medical Research Council framework for evaluating complex interventions (Skivington et al., 2021), the mixed-methods process evaluation uses the Reach, Efficacy, Adoption, Implementation and Maintenance framework (Glasgow et al., 2019) to assess implementation, effectiveness and potential sustainability (Table 3). Quantitative process data will include records on module attendance and completion, as well as facilitator competency measured using the EQUIP-based FamilyACT tool (all facilitators are observed and rated by a trained competency rater at post-training and after completion of the seven core sessions). Supervisors will directly observe two sessions and complete observational ratings assessing fidelity to the intervention manual and quality of delivery. Regular debrief meetings will be held throughout implementation to document challenges. Debrief notes and qualitative data from semi-structured interviews and focus group discussions with families, facilitators and other stakeholders will be analysed using thematic content analysis (Braun and Clarke, 2025). Process and effectiveness data will be triangulated to better understand which families are most likely to drop out or benefit from the intervention and to explore mechanisms of change.

### Trial and adverse events monitoring

A trial management committee will monitor the implementation of study procedures and protocol deviations. All research staff will follow War Child’s Child Safeguarding Policies and will record any suspected maltreatment, exploitation or other violations and report these cases and other adverse events within 24 hours for review and appropriate action. To manage participant distress, multiple mitigation measures will be in place: (1) research assistants will be trained to recognise signs of distress and remind participants they can skip questions or withdraw at any time during all assessments; (2) referral pathways have been established for families requiring support beyond the scope of the intervention, including those who experience distress during intervention sessions or where severe mental health or protection concerns become apparent; (3) all staff have been trained to refer cases in accordance with safeguarding policies. A Data Safety Management Committee will also be established and mandated to provide oversight on adverse events and safety protocols for the trial.

### Ethics

Ethics approval was obtained from the University of Jordan (ref: 557 / 2025). The core research team also comprises representatives from academic institutions and implementing partners who work closely with the target communities. The team will provide input on study materials, implementation approaches and interpretation of findings to ensure cultural appropriateness and relevance.

Particular attention will be given to the ethical considerations of working with vulnerable populations, including refugees and displaced families. The research team will ensure that participation is voluntary, informed consent procedures are clear and accessible and participants understand their right to withdraw at any time without consequences for their access to services. Families will not receive compensation for their participation in the study; they will only be compensated for transportation costs. Data protection measures will safeguard participant confidentiality, and referral pathways will be established for cases requiring additional support.

### Trial status

Recruitment of study participants will begin in December 2025.

## Discussion

This study aims to demonstrate the impact of an integrated approach to family mental health support for displaced and vulnerable families. It tests both upstream family mechanisms and direct impacts on adolescent mental health, providing evidence for family-systemic intervention pathways. The results will contribute to the evidence base on whole-family mental health interventions in humanitarian settings, an area currently characterised by substantial gaps, despite promising findings from caregiver-focused programmes (Giusto et al., 2025). Such integrated approaches could promote sustainability and scale in humanitarian and low-resource settings (Troup et al., 2021).

Study strengths include rigorous evidence on interventions addressing social determinants of mental health through inclusion of both displaced and host populations, use of non-specialist facilitators following task-sharing approaches (Raviola et al., 2019; Cohen and Yaeger, 2021) and modular design tailoring to family-specific needs (Kazdin, 2019).

Limitations include the complexity of measuring change in multiple outcomes over time through the sequential dual outcomes approach. While our sample size provides adequate power for testing H1 (upstream mechanisms), we acknowledge that H2 and H3 are positioned as exploratory analyses. Findings from these exploratory analyses will inform future adequately powered mechanistic studies. Additionally, while the intervention addresses multiple family stressors, some structural challenges facing refugee populations will lie beyond the intervention’s scope. Finally, we will use caregiver-report of child mental health challenges for the screening, as this is most feasible in real-world settings. While this approach has been validated in other Arabic-speaking populations (Ababneh and Alomari, 2016; Emam et al., 2016), adult proxy-report of the SDQ could result in underreporting of internalising symptoms compared to adolescent self-report. Nonetheless, adolescent-report SDQ will serve as the primary, upstream effectiveness outcome and both adolescent- and caregiver-report SDQ will be measured at all time points. Research assistants are also trained to report observations and disclosures of severe mental health and protection concerns as part of consent, screening and all rounds of data collection, ensuring referral to specialist support for respondents and their families.

## Supporting information

10.1017/gmh.2026.10134.sm001 Blackwell et al. supplementary material Blackwell et al. supplementary material

## Data Availability

Data may be available upon request from the corresponding author, subject to ethical approvals.

## Footnotes

1 This differs from Level 2 interventions, which typically involve community-based psychoeducation or peer support groups for families, and Level 4 interventions, which require specialised clinical services such as family therapy delivered by mental health professionals.

2 Sample size calculations were conducted using G*Power (version 3.1.9.7) and based on one index adolescent per family for the primary outcome assessment. In families with more than one adolescent meeting inclusion criteria, the index adolescent will be the child who scores highest on the mental health screening measure.

## Supplementary material

The supplementary material for this article can be found at http://doi.org/10.1017/gmh.2026.10134.
