# Supplementary material for: A family-systemic intervention for mental health with refugees in Jordan: Protocol of a randomised controlled trial of StrongerTogether
Source: Glob Ment Health (Camb). 2026 Feb 4;13:e38. doi: 10.1017/gmh.2026.10134 (PMC12951342; doi:10.1017/gmh.2026.10134)
Supplement: Blackwell et al. supplementary material [file S2054425126101344sup001.docx]

**RA name: _________________ Date: __________________**

**Family Code: ______________**

|  | **ID** | **Total scores** | **Threshold (yes/no)** |  |  | **ID** | **Total scores** | **Threshold (yes/no)** |
| --- | --- | --- | --- | --- | --- | --- | --- | --- |
| 1) | K10 parent ID: | Total score: | 20 or more? Yes / no |  | 1) | K10 parent ID: | Total score: | 20 or more? Yes / no |
|  |  |  |  |  |  |  |  |  |
| 2) | SDQ child ID: | Total score: | 17 or more? Yes / no |  | 2) | SDQ child ID: | Total score: | 17 or more? Yes / no |
|  | SDQ child ID: | Total score: | 17 or more? Yes / no |  |  | SDQ child ID: | Total score: | 17 or more? Yes / no |
|  | SDQ child ID: | Total score: | 17 or more? Yes / no |  |  | SDQ child ID: | Total score: | 17 or more? Yes / no |
|  |  |  |  |  |  |  |  |  |
| 3) | Parent ID like above | Family relationship challenges | Yes / no |  | 3) | Parent ID like above | Family relationship challenges | Yes / no |
| 4) | Parent ID like above | Parenting challenges | Yes / no |  | 4) | Parent ID like above | Parenting challenges | Yes / no |
| 5) | Parent ID like above | Financial challenges | Yes / no |  | 5) | Parent ID like above | Financial challenges | Yes / no |
| 6) | Parent ID like above | Parent: Risk Assessment | Yes / no |  | 6) | Parent ID like above | Parent: Risk Assessment | Yes / no |
| 7) | Parent ID like above | Parent: previous MHPSS Level 3 / 4 | Yes / no |  | 7) | Parent ID like above | Parent: previous MHPSS Level 3 / 4 | Yes / no |

**Eligible** Family need to meet **at least 1 out of 2 criteria:**

1. **At least one parent scores >=20 on K10**
2. **At least one child scores >= 17 on SDQ (only one parent to report)**

**And at least 1 out of 3 criteria:**

1. **Family relation challenges causing significant distress = yes**
2. **Parenting challenges causing significant distress = yes**
3. **Financial challenges = yes**

**And all:**

- **No to risks / safety concerns (parents) – See #6 “risk” above**
- **No to safety concerns (for the child) – RA would inform you, part of assent and bassline with child**
- **No previous psychosocial or mental health support or services for their wellbeing to address severe stress, parenting, or financial stress – See #7 “previous MHPSS Level 3/4” above**
