# Supplementary material for: A family-systemic intervention for mental health with refugees in Jordan: Protocol of a randomised controlled trial of StrongerTogether
Source: Glob Ment Health (Camb). 2026 Feb 4;13:e38. doi: 10.1017/gmh.2026.10134 (PMC12951342; doi:10.1017/gmh.2026.10134)
Supplement: Peer review history

# Reviewer report: Review: A family-systemic intervention for mental health with refugees in Jordan: Protocol of a randomised controlled trial of StrongerTogether — R0/PR2

**DOI:** 10.1017/gmh.2026.10134.pr2

Thank you for the opportunity to review this trial protocol. The authors make a compelling case for the importance of this study and the need for further evidence on family systemic interventions in humanitarian settings. The manuscript is clear and well-written, and the trial builds on previous work that has already demonstrated the feasibility and acceptability of the intervention.

I will first summarize major comments/concerns about the manuscript before detailing specific points.

1. The introduction highlights the need for integrated, holistic interventions that simultaneously address mental health symptoms as well as their social determinants - namely economic hardship. This is an incredibly important point and a significant gap in the field. However, the intervention only includes one optional module focused on financial literacy (i.e. learning how to identify financial needs, create budgets, and save). While I fully appreciate the difficulty in addressing the structural or root causes of economic hardship through a psycho-social intervention, it is a stretch to frame this intervention as addressing social and environmental determinants because it includes one optional module on financial literacy. The focus on financial skills implicitly assumes that the financial challenges faced by families are due in large part to lack of knowledge and skills, rather than the very real problems of lack of jobs, legal documentation, income, aid, etc. Given the extreme vulnerability of the target population, it may come across as insensitive and unhelpful to be offering financial literacy training and it is possible there will be little uptake if the training is not accompanied by improved access to concrete support. I would suggest reframing the introduction to avoid implying that the intervention will address social and environmental determinants of mental health, and acknowledge earlier and in more detail that the intervention does not address these determinants.

2. The evaluation of the ReachNow detection tool and FamilyACT is an important component of this study. However, even though this is stated as a secondary research objective, there is insufficient information on the tools themselves and how they will be evaluated and validated. The hypotheses state that the “ReachNow tool will accurately identify 70% or more of families in need of focused mental health support as identified through clinical assessments.” Was the ReachNow tool included in the previous feasibility study and if so, how did it perform? How does the decision algorithm work? Who will conduct the clinical assessments, what will they consist of, and will they be with the whole family? Similarly, what methods and criteria will be used to demonstrate adequate reliability and

validity of FamilyACT for assessing facilitator competencies in delivering family-systemic interventions?

3. More information is needed on the screening procedures and items. Was the screening tool previously assessed in the feasibility study and if so, how did it perform? How were cut offs for the screening items determined? Have these cut offs been validated for this study population? If not, what are the implications/limitations of this? It looks like screening for child mental health challenges is based on caregiver report only. It’s important to consider the limitations of this approach - namely, incongruence between caregiver and adolescent reports particularly around internalizing symptoms. Given that primary effectiveness for the adolescent mental health outcome will be based on adolescent reports, is there any possibility of screening adolescents directly for this outcome?

Finally, one of the exclusion criterion is elevated screening scores indicating severe mental health concerns. How will you differentiate between those with elevated scores indicating appropriateness for the intervention versus those with mental health concerns that are too severe for this intervention?

Other points are detailed below:

- Typo on page 5, line 28 “facilitatory”

- Page 7, line 20 - should this read “one or both caregivers”?

- Clarify that the intervention is delivered to individual families not to groups of families

- Page 8 exclusion criteria: clarify if all family members or only all participating family members need to give consent. Also clarify if families with any participating member receiving level 4 interventions will be excluded? In some cases, the line between level 2 and level 3 interventions is blurred. How will you determine if the intervention that a family member is receiving does not meet the definition of a level 3 intervention?

- There is no information on whether families will receive compensation for participating in the study and if so, what the value of compensation will be and how/when compensation will be provided.

- Sample size: the authors state that the sample size calculation was based on “most stringent” test involving the upstream primary outcomes. It seems instead that the sample size should be calculated based on H2 and H3. Not all index adolescents will have elevated baseline distress so the study may be underpowered to test H2. Similarly, the sample size calculation does not detail if the study is powered to test moderation effects in H3.

- Page 9: as stated above, please describe how ReachNow performed in the feasibility trial and include more information on the decision algorithm.

- Page 10: Masking: how will level of (un)masking be assessed?

- Outcome measures: for all measures, please state if caregiver and/or adolescent reports will be collected and which will be used as the primary report.

- Page 13: the table does not include any information (besides citation) on caregiver reported SDQ

- Page 14: consider using a different word for “community gatekeepers”

- Please provide more information on how implementation fidelity will be assessed. Will there be fidelity monitoring tools used? Who will conduct fidelity monitoring/assessment? Module completion does not adequately assess fidelity.

- As stated above, since evaluation of ReachNow and FamilyACT are included as secondary objective, there needs to be more information on methodology in the narrative not just in the table.

- Page 16: adoption is focused on facilitators. Will there be any interviews conducted with implementing organizations staff (e.g. managers) and other stakeholders?

# Reviewer report: Review: A family-systemic intervention for mental health with refugees in Jordan: Protocol of a randomised controlled trial of StrongerTogether — R0/PR3

**DOI:** 10.1017/gmh.2026.10134.pr3

**Authors:** Jaikumar C

**Affiliations:** NIMHANS, India

A well conceptualized study, aiming at enhanced holistic family functioning. Strength of family wellbeing as a contributor for individual wellbeing in humanitarian context is often ignored, this study could effectively address the gap. My compliments to all authors and publisher

Please Clarify/ Add:

1. What would be the size of the families recruited for the study? Any optimal number of members?

2. When we say holistic family systemic intervention, it is important to address the needs of all the members. The study objectives and the hypotheses seem to restrict itself only to adolescent wellbeing (which may be reflected on the title of the study) Parenting practices and its impact may be measured in children from across developmental stages and can be mentioned as an indicator to substantiate the comprehensive nature of the trial.

3. What are the ethical measures planned towards distress redressal mechanisms for families who undergo sessions during the study period?

Methodology

1. How the (line number 34 or 35) handout or audio recording will be carried. For handout distribution, it is expected that the participant should have the necessary education level. If, Audio recording is done, how it will be done, is it participants will do the audio recoding or someone from the research team will do the same. Please clarify the same.

2. After adjusting for the attrition rate, final sample size should be 550 and not 528.

3. Two groups ETAU and the intervention are almost homogenous and how the ICC is assumed to be 0.02.

4. It is not clear how the population for this study with inclusion/ exclusion criteria will be identified. How they will be selected. If, it is assumed to be purposive sampling, even then also it is not clear. How many will be identified and selected, and what % is expected to give consent to participate in the study. This can be addressed for the end users understanding.

5. Measuring stress with displaced population will vary with time of the event, how it will be accounted.

# Editor report: Recommendation: A family-systemic intervention for mental health with refugees in Jordan: Protocol of a randomised controlled trial of StrongerTogether — R0/PR4

**DOI:** 10.1017/gmh.2026.10134.pr4

**Affiliations:** Clinical Psychology, National Institute of Mental Health and Neuro Sciences Department of Clinical Psychology, India

Dear Authors,

I am happy to share with you that your manuscript has undergone anonymized review by two independent reviewers, who have provided a comprehensive viewpoints and have recommended for a major review. I, too, have read the reports and found them very useful to enhance the scope of the study. Therefore, I request you to consider addressing the queries and suggestions and resubmit the manuscript.

Thank you for choosing the Cambridge Prisms: Global Mental Health to showcase your work.

Best regards,

Thomas

# Editor report: Decision: A family-systemic intervention for mental health with refugees in Jordan: Protocol of a randomised controlled trial of StrongerTogether — R0/PR5

**DOI:** 10.1017/gmh.2026.10134.pr5

**Affiliations:** London School of Hygiene & Tropical Medicine, United Kingdom of Great Britain and Northern Ireland

No accompanying comment.

# Reviewer report: Review: A family-systemic intervention for mental health with refugees in Jordan: Protocol of a randomised controlled trial of StrongerTogether — R1/PR7

**DOI:** 10.1017/gmh.2026.10134.pr7

Thank you for the thoughtful revisions to the manuscript. There are only two inconsistencies that need to be addressed.

- Please confirm that accuracy of the ReachNow tool will only be evaluated by comparing against screening results, and not by clinical assessments conducted by a psychologist. The clinical assessments by a psychologist are included in the response to reviewers document and on page 5 of the revised manuscript under Primary implementation hypotheses; however, it was removed from page 10 of the revised manuscript.

- Also on page 5 of the revised manuscript under Primary implementation hypotheses, the authors state that the FamilyACT tool will demonstrate adequate reliability and

validity for assessing facilitator competencies in delivering family-systemic interventions in

humanitarian settings. However, the statement on how reliability and validity will be assessed and how “adequate” will be defined was removed from page 14 of the revised manuscript. Please explain how the FamilyACT tool will be evaluated.

Please add to the section on limitations the potential implications of using caregiver report only to screen for adolescent mental health difficulties.

Thank you for the opportunity to review this manuscript again. This is an important study.

# Editor report: Recommendation: A family-systemic intervention for mental health with refugees in Jordan: Protocol of a randomised controlled trial of StrongerTogether — R1/PR8

**DOI:** 10.1017/gmh.2026.10134.pr8

**Affiliations:** Clinical Psychology, National Institute of Mental Health and Neuro Sciences Department of Clinical Psychology, India

Dear Authors,

I am happy to share with you that the independent review is positive but have suggested a minor review that amounts to clarifying a few points. For more details, please see the reviewer’s comments. I hope you will find the review useful and submit the revised manuscript at the earliest.

Best wishes,

Thomas

# Editor report: Decision: A family-systemic intervention for mental health with refugees in Jordan: Protocol of a randomised controlled trial of StrongerTogether — R1/PR9

**DOI:** 10.1017/gmh.2026.10134.pr9

**Affiliations:** London School of Hygiene & Tropical Medicine, United Kingdom of Great Britain and Northern Ireland

No accompanying comment.

# Editor report: Recommendation: A family-systemic intervention for mental health with refugees in Jordan: Protocol of a randomised controlled trial of StrongerTogether — R2/PR11

**DOI:** 10.1017/gmh.2026.10134.pr11

**Affiliations:** Clinical Psychology, National Institute of Mental Health and Neuro Sciences Department of Clinical Psychology, India

Dear Authors,

On behalf of the journal, I appreciate your time and efforts to revise and submit the manuscript. I look forward to your support to the journal in quality research for global mental health.

Best wishes,

Thomas

# Editor report: Decision: A family-systemic intervention for mental health with refugees in Jordan: Protocol of a randomised controlled trial of StrongerTogether — R2/PR12

**DOI:** 10.1017/gmh.2026.10134.pr12

**Affiliations:** London School of Hygiene & Tropical Medicine, United Kingdom of Great Britain and Northern Ireland

No accompanying comment.
